# Essai sur la Neuralgie du Grand Sympathetique

**Published:** 1838-10-01

**Authors:** 


### ESSAI SUR LA NEURALGIE DU GRAND SYMPATHIQUE, MALADIE CONNUE SOUS LES NOMS DE COLIQUE VEGETALE, DE POITOU, &c. &c. Par *A. Segond*, D. M. P. Paris, 1837.

ALTHOUGH perhaps the author arrives rather too hastily at the conclusion, that the species of colic he here describes is essentially a neuralgia of the sympathetic nerve; yet has he produced an excellent monograph upon the subject, a brief analysis of which will probably be acceptable to the readers of this Journal.

The "Vegetable Colic" is that form of the disease, which has been described under various names, as prevailing in different parts of the world. It has often been named from its locality, as Poitou, Devonshire, Madrid, Surinam Colic, &c.; or from some remarkable feature, as bilious colic, colic from depraved secretions, colic from injurious ingesta; and is the same disease as the hepatic ileus, or dry belly-ache of the West Indies, described by Dr. Musgrave. (*London Med. Repository*, 1823.) The term it is generally known by in France is "Colique Vegetale"—a name, as the author observes, obviously improper, since vegetable ingesta take no part in the production of the disease. It prevails to a great extent in the inter-tropical regions; and Dr. Segond, as chief medical officer of the French Marine at Guayana, had ample opportunities of observing it there.

*Causes and Predisposition.* Dr. Segond entirely rejects the idea of its being caused by unwholesome fruits, vegetables, ill-fermented drinks, &c. &c.; although he admits that these may determine an attack in persons predisposed to it by any epidemic cause. If, as is generally supposed, the above ingesta could produce the disease, we should not find women and children entirely exempt, and persons fall victims to it who partake only of the most esteemed diet; while again the marine service, although continuing precisely the same diet which secured health elsewhere, becomes attacked on approaching places where it is known to prevail at certain seasons. To attribute it, as is sometimes done, to the poison of lead, derived from the water-pipes, adulteration of wines, &c., is equally erroneous; for while, where precautions have been taken against these causes, the disease has nevertheless appeared, on the other hand, where such causes have universally existed, in other places, no similar affection resulted: moreover, the colic from lead is attended with certain distinctive symptoms, as we shall see hereafter. The substances he most mistrusts are alcohol and coffee; and the reader will recollect, that Dr. Musgrave attributed the affection to the large quantities of rum consumed. Climatology would seem to afford the true key to its production, atmospheric influences often prevailing epidemically, being, according to Dr. S.'s experience, its true source. Thus, it has been found to prevail especially in places where the contrast of temperature, by day and night, is marked, and where the vicissitudes are sudden, as in the elevated parts of Spain, and numerous inter-tropical situations. Again, at Guayana it varies in the most remarkable manner, according to the season; and is either not present at all, or is very mild, during the equable heats of Summer, attacking chiefly the new comers, who rashly expose themselves ill-clad to the night-air; while during the Winter, and especially when a cold wind blows from the North, it becomes intense, and often violently epidemic. Any cause tending to check transpiration, powerfully aids the development of the disease, and thus it was frequently found to supervene upon exposure to cold currents of air while heated or sleeping, the taking cold beverages while hot, &c. &c. Persons, too, whose occupations or wants involved an exposure to sudden vicissitudes of temperature, were found exceedingly liable to be attacked. Thus sailors, fishermen, collectors of natural history, soldiers, bakers, cooks, blacksmiths, &c., and those persons who were too poor to obtain sufficient food and night-lodging, furnished nearly all the examples of the colic at Guayana. It attacks only the male sex, and seldom the two extremes of life. Persons of a bilious temperament, and of a nervous sensitive constitution, are eminently liable; as also are those suffering from nostalgia and melancholy, or who have debilitated their constitutions by alcoholic drinks or excessive venery—too common in these regions.

*Symptoms.* The author describes these in a very clear, and detailed manner; the following is a summary of the principal ones—there exists, for several days, a preliminary sense of *oppression* and *discomfort* (malaise abdominal) in the abdomen, accompanied with a cast-down icteric *countenance,* highly expressive of the uneasiness felt, and speedily followed by inertia of all the functions of organic life, and general corporeal debility. The *abdomen* soon becomes excessively distended by flatus, but unattended with tenderness, unless gastro-enteritis be present, which it very rarely is at the commencement Frequent *vomiting*, at first, only of the contents of the stomach, but afterwards of a yellow or greenish, acid, bilious fluid. The *stools* which, at first, are frequent and loose, soon become infrequent, dry and hard, and after awhile are succeeded by complete costiveness. From four, to five or six days may elapse, before the *colic-pains* occur; attacking, first, the duodenic, and, afterwards, the hypogastric region; they are of an intolerably lacerating character. The *pulse* is slow and unequal, but may be entirely natural, if no inflammatory complication exist. *Tongue* at first white and moist, becomes dry, as do the fauces, but there is little *thirst. Temperature* varies. The *urine* becomes scarce, and disordered in quality, whence great vesical irritation ensues. After awhile, great pain is felt in the region of the kidneys, along the spine, and in the extremities and trunk; when it invades the latter, the inspiratory muscles are sometimes nearly prevented acting, producing a state of the most imminent danger. The general debility increases, and the limbs become paralysed or convulsed. After several days, symptoms of decided fever, accompanying excessive abdominal tenderness, often manifest the co-existence of a *gastro-enteric* affection. Finally, the *Cerebrum* sympathising with, or participating in, the general disorder of the economy, we have delirium, general convulsions, perversion of the senses and intellects, &c. &c.

It is rare, however, for the disease to pursue this severe course, if judiciously treated. Its duration will much depend upon the season, and the prevalence of an epidemic state of the atmosphere; but, as a general rule, (although in some rare cases, promptly treated, it may be removed in a few days), it is a most tedious malady, having a greater or less number of *remis-*

*sions* in its progress, giving it somewhat the character of an intermittent disease, although it is totally unaffected by the remedies appropriate for that class of affections. It is almost impossible, by any treatment, to prevent frequent *relapses* in the course of the cure, even when the inexperienced would consider the patient quite secure, unless he be removed from the endemic influences which surround him. Where good alvine evacuations, accompanied by a plentiful secretion of healthy bile, continue for a fortnight, we may begin to be tolerably certain of a favorable termination. When the disease passes into the *chronic* state, accompanied as it usually then is, by low fever and other signs of gastro-enteritis, it is one of the most deplorable and hopeless of maladies. The patient wasting away, his limbs atrophied, palsied or trembling, his intellect impaired or perverted, can only linger out a wretched existence.

*Anatomical Characters.* Dr. Segond laments the paucity of anatomical observations on this disease. Almost all writers have set it down, either as a gastro-enteritis, or as a purely nervous affection. He himself considers that there is, in the first place, functional lesion of the sympathetic only, but that, in the progress of the disease, the organs it supplies become deranged in their operations, and may sometimes develop inflammatory action; but he adds, "as during life we must learn to distinguish a disease from the complications which supervene, so after death, must we trace the distinction between the principal or fatal lesions, and those which are accidental or secondary." It being a disease seldom fatal, he had but few opportunities of examination after death, and only relates the result of two in his book; which, however insufficient they may be to establish the doctrine of the nature of the disease he advocates, are very interesting as far as they go. We present an abstract of the appearances of the sympathetic. In the 1st case:—"The ganglia of the abdomen of a reddish-brown or yellowish colour, (resembling agate), and hard as if hypertrophied; their branches of communication more apparent and voluminous, than in the normal state; on the right semi-lunar ganglion is a yellow spot, not penetrating its substance entirely. The solar plexus is increased in volume, the filaments and ganglia entering into its composition, having lost their natural softness, resist and creak under the scalpel, the ganglia themselves being of a brownish colour, spotted with yellow. The plexuses, also, which emanate from the solar, are larger, more prominent, and more easily traced than in the normal state." p. 28.—In the 2d case:—"All the ganglia are in a morbid state: thus, those of the chest are very large, and of a vivid red, spotted here and there with yellow, and their branches more apparent than in the normal state. The swollen ganglia of the abdomen present a yet more unusual volume, their redness not being so great, but their density greater than the thoracic. The semilunar ganglia are also encreased, and cartilaginous. The solar plexus is equally enlarged, and its density so much encreased that it is hard when cut into, while its secondary plexuses have undergone like changes. To be certain that the characters we observed, were truly pathological, we took an opportunity which presented itself, of comparing them with those of two bodies in the theatre, the one an adult, the other a child eight years of age." p. 35.

Dr. Segond refers to M. Pascal's experience, without, however, detailing it: we may mention, that this physician treated the colic at Madrid, and in the Recueil de Memoires de Med. et de Chir. Mil. vol. 19, 1826, relates, that he found the ganglia diseased in several cases he examined, and to their communications with the spinal nerves, he attributed the paralysis of the limbs.

We may here remark, how lamentably deficient is our knowledge of the pathology of the sympathetic nerve, even in diseases upon whose nature it might throw great light. The hurried and unsatisfactory manner in which "post-mortems" are conducted generally in this country, is much to be regretted;—rare indeed is it, that this system is made the subject of enquiry at all. We venture to entreat those medical men, to whom frequent opportunities occur, to neglect this important subject no longer; and in order to show with what facility the examination may be accomplished, we transcribe Mr. Swan's directions for that purpose:—

"In *every examination* of the human body, the state of the par vagum and grand sympathetic nerve may be *readily* observed, if the body be opened in the following manner:—An incision being made through the skin from the middle of the neck to the symphysis, and the skin turned back, the pectoral muscles are to be raised from their origins. The abdomen is to be opened through the linea alba, and then the attachment of the abdominal muscles to the ribs are to be divided as far as convenient. The clavicles are to be sawn through about their middle, and the ribs divided near their angles.* It is better to begin at the lowest rib, and divide two or three on each side, and then separate the diaphragm as far as it is attached to them. When the sternum and ribs attached to it have been removed, the viscera of the chest and abdomen are to be examined in their situations. After this *every drop of blood is to be washed away* with a sponge and water. The pleura may then be stripped from the ribs by taking hold of it at the part where they were divided, and turning it back towards the posterior mediastinum, when the nerves may be very readily dissected with the forceps and one blade of a pair of scissors. In making the examination, it is absolutely necessary to avoid wounding the veins, and if it be absolutely necessary to remove any of them for the purpose of seeing the parts behind them, then, two ligatures must be placed on each side of them before a division is made, as it is impossible to draw such satisfactory conclusions from the appearances when the nerves have been covered with blood."—*Enquiry into the Action of Mercury*, 2nd Ed. pp. 34, 35.

The *Treatment* advocated by our author, we think highly judicious, and he informs us it was very successful. The grand desiderata he considers, are "to relieve the pain, restore due evacuations, and guard against inflammation." In regard to the *pain*, its relief requires that we should be fully aware of the true nature of the disease;—that is to say, we must not treat it as *inflammatory*, but, as resulting from a perverted state of innervation, which has secondarily disordered the functions of several organs, an early effect of such disorder being to produce a degree of depression or torpor in them. Thus if we employ debilitating measures, and apply large doses of opium so near the seat of evil, as is the gastro-intestinal mucous membrane,

* In dividing the ribs, Mr. Swan employs a pair of scissors "similar to those used by gardeners, in pruning trees: one blade is narrow and very thick, and is concave at the edge;—the edge of the other is convex, broad, and much thinner." With these the bones are divided with ease and expedition.

although perhaps we do obtain some temporary alleviation of the pain, it is by inducing additional torpor and debility in organs already too inactive and depressed. By applying *blisters* on each side of the spinal column, we break through this anomalous state, and then we can with more propriety have recourse to *opium*, but it is much better to apply it in the form of *morphine* to the blisters, taking care, however, that the quantity used be not excessive: the blisters need not be confined to the lumbar region, but may also be applied to the shoulders, back of the neck, pole of the head, &c. according to whether the respiration, voice, or encephalon be involved. When the pain is somewhat relieved, we should be cautious as to continuing the morphia, lest we render the bowels less obedient to the action of evacuants.

To procure *evacuations*, and stimulate the secretory powers of the disordered organs, are most important indications. *Emetics* although so often of the highest use in persons of a bilious temperament, where the bile continues to be secreted and accumulated in the duodenum and stomach, as evinced by great nausea or frequent vomiting, are hurtful in eminently nervous temperaments. *Purgatives*. As the presence of bile determines us as to the use of emetics, so must its absence determine us as to the kind of purgative, since in this case the strongest will not act unless aided by chologogues. Dr. S. praises, as the best means of giving activity to this secretion, a powerful dose of *calomel, aloes* and *soap*, and if this combination be rejected by the stomach, it should be administered in a small quantity of liquid, containing some opium, as an injection. These medicines will usually procure some bilious stools, but efficient purging will not result until some saline purgative has been added. As soon as this occurs we should be careful to discontinue the calomel. In some cases, which have resisted these means, he has employed frictions of *croton oil* (diluted with castor oil), but he has not given it internally, fearing the *poisonous* effects of large doses. This reminds us of a case, which once fell under our notice, where, to relieve constipation consequent on hernia of old standing, croton oil was given in doses of two or three drops, every two or three hours, until it operated. The purging which resulted was not immoderate, and the symptoms were relieved, but the patient without assignable cause gradually sank; and as no reason could be discovered for this after death, we concluded he died from the poisonous effects of this medicine on the nervous system. If we recollect aright, he took about a dozen drops in all: he was a man of good, though not robust constitution, aged between 50 and 60, and very temperate. Where the constipation is obstinate, the author passes great encomia upon the decoction of *fresh tobacco leaves*. He gives two or three lavements, if no symptoms of narcotism are present, and has never known the bowels resist.*

* The reader will remember Dr. Abercrombie's opinion upon this subject: "The tobacco injection, as far as my observation extends, is the remedy of most general utility in all the forms and stages of Ileus. It should be given, at first, with much caution, perhaps not more than 15 grains infused for ten minutes in 6 ozs. of boiling water. After the interval of an hour, if no effect be produced, it may be repeated in the quantity of 20 grs. and so on, until such effects are produced, in slight giddiness, and muscular relaxation, as show that its peculiar action is taking place upon the system. It may then be repeated, at intervals of

Various subordinate remedies (as baths, fomentations, &c.) must not be neglected, and as *relapses* are characteristically frequent in this disease, the strictest attention to diet and regimen should be enjoined, and above all things, when the health is sufficiently re-established, a change of locality. The purgatives must be continued, and the urinary secretions encouraged by diuretic drinks, while the surface of the body must be carefully protected by judicious clothing. Where the abdominal uneasiness persists, as also the pains of the limbs, the external and internal use of *turpentine* is often very useful. Although, as has been observed, this disease often almost approaches to an intermittent, in the frequency and duration of its remissions, yet is it not benefitted by the usual anti-periodic medicines, as iron, quinine, &c. As a general rule we shall be more successful in the judicious and modified continuance of the measures useful at the commencement of the disease, than in resorting to the employment of other medicines; however useful these may be in disorders apparently analogous. In regard to the treatment of some particular symptoms: if the *epilepsy* be not attended with symptoms of debility, especially by paralysis of the limbs, bleeding will be useful. For the *paralysis* which may arise, either from the long-continuance of the disease, or from a too active primary treatment, the general and local employment of turpentine is useful; but of all remedies, in this case, strychnine has been the most serviceable—an alcoholic solution being at the same time rubbed upon the spine, and the paralysed parts. The *dyspnœa,* arising from the diminished powers of the inspiratory muscles, is an alarming symptom, and should be met at once with a blister, dressed with strychnine; awaiting the action of which, stimulating frictions should be employed upon the chest and fluids of the same nature swallowed. The *trembling of the limbs* is best relieved by the baths of the sulphuret of potass.

*Antiphlogistic and Debilitating Agents.*—"The symptoms teach us these should not be our first means, although some practitioners see in them our only resources: we have often known their use attended with negative and deplorable results." Should, however, inflammation precede, or suddenly complicate vegetable colic, vigorous antiphlogistic means must be employed. As to general bleeding it is rarely indicated, since this complaint rarely shows itself in the plethoric, and the inflammation of the digestive organs yields better to leeches. But, if in the progress of the neuralgia the cerebro-spinal system becomes seriously involved, we should abstract blood generally, but yet with a due regard to the strength of the economy. It is rare indeed that general bleeding can be required to *precede* in the treatment, and when, in its course, its necessity becomes apparent, it is from some grave complication, or epidemic peculiarity. We are naturally anxious to combat the severe local pains with leeches, but sinapisms to the parts will often be found more useful. The symptoms of vesical irritation, however, require, and are relieved by leeches.

Dr. Segond next relates, as illustrative of the symptoms and treatment, 21

---

one or two hours, a great many times, if the case do not yield speedily, and with the precautions now mentioned, I have never seen any unpleasant effect result from the free use of this powerful remedy."—*Pathology of Intestinal Canal,* p 144.

cases, which he has selected from the great number attended. They are well, and apparently impartially, related.

*Essential Nature of the Disease and its Diagnosis.*—In this section of his work the author, after taking a recapitulatory view of the effects of those causes, (*e. g.* climate, temperature, &c.) which he believes engender an inordinate susceptibility in the organs of vegetative, organic, or internal life, developed as are their functions by these causes into an anormal activity, next reviews several of the phenomena of the disease, and from a consideration of these and the circumstances in which they differ from some analogous diseases, arrives at the conclusion that this system is solely, or chiefly, or at all events primarily affected, and that the various other organs of the body, when invaded by diseased action, are so only secondarily, and as a consequence of the depravation of their secretions by the disorder of the system which regulates them—or from an extension by sympathy of this disorder to the other nervous cavities. Some of his observations we proceed to notice.

*Nature of the Pain.*—In its character it neither resembles the iliac passion, the nervous colic, or inflammation of the mucous membrane. It has a different pathological modification: radiating from the ganglionic system as from a centre, it extends to the various abdominal organs, the limbs, bladder, &c. It is of a twisting, tearing, and lacerating nature, apyrectic, internal, and unincreased by pressure. Its extent, its duration, and the means by which it is relieved, equally show its non-inflammatory nature.

*Nature of the Treatment.*—The treatment we have described, could not have been successful had this been a primary inflammatory disease. We have found that antiphlogistics are not the best means of combating it; and yet the too free and indiscriminate use of opium is not admissible, for although the pain be nervous, we must not add to the physiological depression and inertia of the implicated organs.

*Distinction from Bilious Colic.*—The author defines this, as colic arising from the presence of biliary calculi, which is certainly sometimes an accidental complication also in vegetable colic. But in bilious colic, stools are present, and there is inflammatory action. In vegetable colic, the liver in common with the other digestive organs suffers more or less, accordingly as it may have been predisposed,—but yet its affection is but secondary, and occasioned by the altered state of its secretory powers, diminishing and vitiating the bile, and sometimes inflaming the mucous passages it traverses. The definition Dr. S. here gives, as the one usually received of bilious colic, there can be no question, is too limited, for although he himself may bestow that name only upon the colic which is caused by calculous accumulations, yet, many other authors believe the liver to be the part primarily affected, and by its suppressed and vitiated secretions, causing a train of symptoms identical with the "colique vegetale." Thus Dr. Musgrove, taking this view of the disease, denominates the dry belly-ache of the West Indies, hepatic ileus, attributes its origin to the effects of rum upon the liver, and treats it with mercury carried to salivation.

*Distinctions from the Lead Colic.*—Although these two affections may be confounded together in books, they cannot at the bed-side. A most important distinction is the sporadic character of the one, and the (almost invariably) endemic or epidemic prevalence of the other. In metallic colic, the patient's health may be sometimes re-established, even without medical aid, which is impossible in the vegetable—at least while he remained in the same locality, preventing as that does, an estrangement from the cause, which may occur in regard to the metallic. In vegetable colic, there is a greater tendency to the inflammatory complication, with accompanying fever and tenderness. The great distention of the abdomen, is one of the best distinguishing marks of vegetable colic, from the metallic, in which, there is great retraction of that part. The countenance, in the lead disease, is natural between the paroxysms, while in vegetable colic, it permanently indicates the oppressed state of the organism. In the latter, there is more marked icterus from the greater participation of the liver in the chain of disordered actions. While suppression of urine is not observed in the colic from lead, the retraction of the testicle, and contracted state of the anus and rectum, found in it, do not prevail in the vegetable colic. Epilepsy is a much more alarming symptom in lead colic, arising as it does from idiopathic disease, produced by the poisonous effects of the metal, while in the vegetable, though a serious superadded phenomenon, it is merely a functional affection. Paralysis of the limbs is less immediate and imminent, but more dangerous and obstinate, in the vegetable colic. The diagnosis is not difficult in the lead colic, but requires experience in the vegetable: the prognosis is favorable in the former, difficult and tedious in the latter. Lastly the treatment of the two maladies differs.

The author concludes his work by examining how far the functions, whose lesions he has described depend upon the sympathetic nerve; and he here entirely bases his observation upon the Researches of Brachet upon the Functions of the Ganglions (recherches experimentales sur les fonctions du systeme nerveux ganglionnaire). For example, as regards the *stomach*, while we see the par vagum establishing the communication with the cerebrum, for the expression of its wants, and for the performance of its movements; the secretion, exhalation and absorption of its fluids are under the direction of the ganglionic system. Thus in colique vegetale, inappetence rather than a disgust at food, is present; chymification becomes imperfect, as the power of producing the necessary fluids is diminished or suspended. The dominant sensation, at first, is rather one of weight or heaviness, than pain, and if vomiting occur, it is not so much from erethism of the stomach, as from the accumulation which has occurred in it, since its absorbing powers have diminished. The lesion, then, affects rather the vegetative, assimilative or chemical life, than the relative life of this organ. Thus too, the exhalation of fluids upon the mucous surfaces of the *intestines*, being almost entirely suspended, we have a deficient chylification, and an increased dryness of the stools, with an almost impossibility of obtaining them; and although this last may, in some measure, arise from the absence of bile, yet after that has been obtained, they continue infrequent, hard, and globular, until the intestinal secretions are freely re-established. In the *rectum*, the sensations are not blunted, but morbidly encreased, being excited, however, not by the presence of fæcal matter, but by the acrid nature of the depraved secretions, exciting the sympathetic action of the spinal nerves. The *kidneys* secrete the urine with difficulty, in small quantities, and of altered qualities. The *bladder* placed like the rectum, under the influence of the spinal nerves. gives rise to very troublesome symptoms; irritated by the nature of its contents, the attempts to evacuate them are urgent and distressing, inducing, sooner or later, a diseased state of the membrane, and requiring medical means to be directed expressly to their relief. In the *lungs*, although the sense of the necessity of respiration, and the mechanism by which it is effected, depend upon the pneumogastric and intercostal nerves, yet the chemical phenomena,—the secretory powers, are subjected to ganglionic not cerebral influence; the respiratory function is affected in the first instance, but it would seem to be so, rather in consequence of the sympathy of the spino-cerebral system with the ganglionic, than from any direct influence of this last itself. The complication is grave, and the danger imminent, when the power of performing efficiently the necessary movements dependent on the cerebro-spinal system, ceases simultaneously with the chemical influences dependent on the ganglionic.

Although Dr. Segond's reasonings are not entirely conclusive, nor his facts sufficiently numerous, he has nevertheless produced a creditable and instructive little work; written as it is in a candid spirit, and with a knowledge of its imperfections. He has studied the disease in a manner essentially clinical, and declares that the theory of its nature, he has broached, was forced upon him, by an attentive consideration of the symptoms, and means of removal. As at the outset of his career, he had no theory to support, or books to consult, his observations are more likely to be unbiassed and trustworthy. The subject is important, and by no means limited to this species of the disease, or the particular climate in which he has observed it; for doubtless whatever throws light upon its essential nature, will, also, more or less, elucidate other diseases of its class. We join him, in entreating all those to whom opportunities may occur, (especially, the officers of the navy and army, to whom medicine already owes so much), to be diligent in the collection of clinical and pathological observations upon this interesting malady.

We may just cite, in conclusion, as somewhat corroborative of the author's view, the observations of *M. Pascal*, who had the opportunity of treating the Madrid colic, and whose pathological experience we have alluded to. He believed it to be a disease situated in the ganglionic system, and found, that the employment of internal medicines to relieve the pain and irritation, and of external revulsives, was far preferable to the obtaining large evacuations by purgatives and bleeding. *M. Marguand*, who treated the same epidemic, and who is said never to have lost a patient, believed in its nervous origin, and treated it by opium and purgatives. *M. Andral*, speaking of the nature of *lead colic*, (and we are far from agreeing with Dr. Segond, as to there being so marked a difference in the *essential* nature of the two diseases, however some of the symptoms and the causes may differ) among other observations, says, "to us it seems the lead colic is a nervous disease, in which there would seem to be especially implicated the spinal marrow, and the abdominal plexuses of the grand sympathetic. The constipation would seem to depend upon, either the annihilation of the contractile movements of the intestines, or upon the suspension of the secretions from their mucous membrane."—(*Clinique Medicale, Tom.* 4).

*M. Ranque*, so well known by his work on lead colic," places the primitive seat of this disease in the portions of the pneumogastric and trisplanchnic which are distributed to the stomach, the intestinal canal, the liver, the kidneys and bladder; and the secondary seat in those portions of the spino-cerebral system, which supply either the painful limbs, or integuments of the head."—(*Journal des Progres des Sciences Medicales, Tom* 2).

The work of *Mr. Teale* on Neuralgia, details several observations on affections of the digestive organs, manifested by pains, altered secretions, and increased flatus, which he attributed to disease of the sympathetic ganglia, and which he found relieved by applications in the neighbourhood of the spine, after the same means had failed, when applied to the seat of pain. He also mentions two cases of colica pictonum, in which the symptoms disappeared after applications to the loins had been resorted to.

